# Prediction of Geopolymer Concrete Compressive Strength Using Novel Machine Learning Algorithms

**DOI:** 10.3390/polym13193389

**Published:** 2021-10-02

**Authors:** Ayaz Ahmad, Waqas Ahmad, Krisada Chaiyasarn, Krzysztof Adam Ostrowski, Fahid Aslam, Paulina Zajdel, Panuwat Joyklad

**Affiliations:** 1 Department of Civil Engineering, COMSATS University Islamabad, Abbottabad 22060, Pakistan; ayazahmad@cuiatd.edu.pk (A.A.); waqasahmad@cuiatd.edu.pk (W.A.); 2 Faculty of Civil Engineering, Cracow University of Technology, 24 Warszawska Str., 31-155 Cracow, Poland; krzysztof.ostrowski.1@pk.edu.pl (K.A.O.); paulina.zajdel@doktorant.pk.edu.pl (P.Z.); 3 Thammasat Research Unit in Infrastructure Inspection and Monitoring, Repair and Strengthening (IIMRS), Faculty of Engineering, Thammasat University Rangsit, Klong Luang Pathumthani 12121, Thailand; 4 Department of Civil Engineering, College of Engineering, Prince Sattam Bin Abdulaziz University, Al-Kharj 11942, Saudi Arabia; f.aslam@psau.edu.sa; 5 Department of Civil and Environmental Engineering, Faculty of Engineering, Srinakharinwirot University, Nakhonnayok 26120, Thailand; panuwatj@g.swu.ac.th

**Keywords:** geopolymer concrete, compressive strength, environment, cement, machine learning, coefficient of determination, fly ash, predictions

## Abstract

The innovation of geopolymer concrete (GPC) plays a vital role not only in reducing the environmental threat but also as an exceptional material for sustainable development. The application of supervised machine learning (ML) algorithms to forecast the mechanical properties of concrete also has a significant role in developing the innovative environment in the field of civil engineering. This study was based on the use of the artificial neural network (ANN), boosting, and AdaBoost ML approaches, based on the python coding to predict the compressive strength (CS) of high calcium fly-ash-based GPC. The performance comparison of both the employed techniques in terms of prediction reveals that the ensemble ML approaches, AdaBoost, and boosting were more effective than the individual ML technique (ANN). The boosting indicates the highest value of R^2^ equals 0.96, and AdaBoost gives 0.93, while the ANN model was less accurate, indicating the coefficient of determination value equals 0.87. The lesser values of the errors, MAE, MSE, and RMSE of the boosting technique give 1.69 MPa, 4.16 MPa, and 2.04 MPa, respectively, indicating the high accuracy of the boosting algorithm. However, the statistical check of the errors (MAE, MSE, RMSE) and k-fold cross-validation method confirms the high precision of the boosting technique. In addition, the sensitivity analysis was also introduced to evaluate the contribution level of the input parameters towards the prediction of CS of GPC. The better accuracy can be achieved by incorporating other ensemble ML techniques such as AdaBoost, bagging, and gradient boosting.

## 1. Introduction

It is clear that concrete material is gaining more popularity as the demand for construction work increases [1]. The demand for ordinary Portland cement (OPC) has also increased due to the rapid surge reported regarding construction work [2]. The concrete sector faces many problems to meet the high demand for OPC due to restricted reserves of limestone, slow production growth, and increasing carbon taxes. OPC is considered one of the predominant building materials investigated in the construction industry and reported as 4.2 billion tons (short scale) production in 2019 [3]. During the manufacturing of OPC, numerous gases affect the environment in series of ways [4]. Acquisition of limestone, an important raw material of OPC, causes water pollution and land pollution and results in the disturbance of the local ecosystem and fauna and flora [5]. The OPC production liberates approximately the same amount of carbon oxide (CO_2_) into the environmental condition and leads to air pollution [6]. CO_2_ emission during OPC production accounts for about 7% of the total greenhouse gas excretion and is responsible for about a 4% enhancement towards global warming [7]. India holds the second position in the world with the annual production of about 502 million tons of OPC and is predicted to be hitting the figure of 550 million tons by 2025 [8]. On the other hand, the disposal of industrial waste such as fly ash ground granulated blast furnace slag (GGBS), silica fume, and other secondary cementitious materials (SCM) also has a serious threat to the environment [9,10,11]. The disposal of these wastes in the water as well as in the land causes water and land pollution, respectively [12,13,14]. The utilization of these industrial by-products to replace the OPC in the concrete leads to another type of concrete named geopolymer concrete (GPC) [15,16,17,18]. Geopolymer materials are alternative binders to OPC cement with a low CO_2_ content. They can be produced by reusing industrial wastes such as fly ash from coal-fired power plants or blast furnace slag. Geopolymer concrete provides improved fire resistance, resistance to chemical corrosion, and enables a viable use of waste materials. The demand and scale of concrete production is a heavy burden for the natural environment. The type and amount of cement being used have the main impact on the size of this load, which is associated with high CO_2_ emissions. The reduction of the concrete burden for the natural environment is achieved by optimizing the composition of the concrete mixture, limiting the cement consumption, by using mixed cements or replacing cement with other binding materials. The most ecological approach is using waste materials as binders that can be used and disposed of in the concrete [19].

Application of SCM in concrete to replace cement content at certain percentages or even at 100% is of great intersect for researchers in the field of civil engineering [20,21,22]. This replacement achieved the desired requirements/strength of concrete and minimized the environmental risks [23]. Ashes from coal combustion in the pulverized-fuel boiler can partly replace the aggregate or cement in the concrete mixture for the production of concrete elements with limited longevity and durability [24]. The ashes after incineration of post-coagulation sediments which contain organic glues can also substitute the aggregate. These wastes, after neutralization attempt by cementation (solidification), can be used as a filler of concrete mixture for the production of prefabricated components [25]. Ashes from the incineration of sewage sludge, after appropriate treatment, can be used as lightweight aggregate or in the production of concretes and mortars, partly replacing Portland cement. These ashes are rich in a phosphorus compound. Therefore, it is assumed that the slow increase of strength of concretes containing such ash may be caused by the presence of phosphate ions, which contribute to delaying the hydration process [26]. Marble waste generated during the exploitation of deposits, by using the shooting technology, can be disposed of as waste marble dust in the production of marble clay bricks and can partially replace cement in air-cured mortar [27,28]. Silica fume is a by-product of the silicon metals production. It is used in the production of additives for cement mortars, industrial concrete, insulation materials, ceramic products, products with an increased resistance to high temperature and high-performance self-compacting concrete [29]. It has been reported that industrial waste and numerous artificial and natural fibers can be successfully utilized to enhance the properties of concrete [30,31,32]. Ganesh et al. [33] research was based on the high-performance fiber-reinforced geopolymer concrete. The glass fibers were incorporated in the GPC to investigate certain properties. It was noted that the energy absorption capacity of GPC was increased tenfold, while the brittleness was decreased significantly. Shahmansouri et al.’s [17] study was based on the application of an artificial neural network (ANN) to forecast the compressive strength (CS) of eco-friendly GPC using silica fume and natural zeolite (NZ) in it. It was reported that the NZ and silica fume have an impressive effect on the CS of GPC. The ANN model also shows the high accuracy of prediction, indicating the R-value equals 0.98 of its training set. Aneja et al. [34] used a type of ANN model to predict the strength properties of fly-ash- and bottom-ash-based GPC. It was reported that the ANN model was effective in the forecasting of GPC strength. Khan et al. [35] studied the application of gene expression programming (GEP) to predict the compressive strength of geopolymer concrete. It was reported that the GEP model is very much effective towards the prediction of compressive strength of geopolymer concrete. Ma et al.’s [36] study was based on both the structural and material properties of GPC. The number of parameters evaluated in the previous studies was reviewed and compared with one another.

Artificial intelligence (AI) evolution in civil engineering plays an impressive role, especially when it comes to predicting the behavior/performance of the materials such as concrete. The use of various machine learning (ML) techniques such as decision tree (DT), ANN, gene expression programming (GEP), AdaBoost, bagging, boosting, support vector machine (SVM), and random forest (RF) are popular for the forecasting of required outcomes [37,38,39,40]. Ahmad et al. [41] used the GEP algorithm to predict the compressive strength of recycled coarse aggregate-based concrete, and it was reported that the GEP model was effective for forecasting the CS of concrete. Song et al.’s [42] research was based on both the experimental evaluation and use of the ANN model for ceramic waste-based concrete. The ANN model was also compared with other DT models and noted that the ANN had a high-performance level of prediction as opposed to DT. Khan et al. [43] used the GEP model to predict CS of fly ash-based GPC. The model’s accuracy was confirmed via statistical checks and external validation. Aslam et al. [44] studied the application of the GEP model towards the prediction of CS of high-strength concrete. The study reveals that the ML approaches proposed adamant accuracy and high-performance level in the prediction aspect. Chu et al.’s [45] research was based on the use of GEP and multi-expression programming (MEP) to predict the strength property of geopolymer concrete. The study also reveals that the GEP model was more accurate in prediction than the MEP model for geopolymer concrete.

This study uses both the individual (ANN) and ensemble (boosting) ML algorithms to forecast the compressive strength of high calcium fly ash-based geopolymer concrete. The ANN and boosting approaches were incorporated for the prediction aspect. The evaluation of the errors, mean absolute error (MAE), mean square error (MSE), and root mean square error (RMSE) were the part of this study, which confirms the model’s accuracy. The statistical checks and k-fold cross-validation process was also adopted to confirm the model’s accuracy. In addition, the sensitivity analysis was also carried out to evaluate the contribution of all input parameters towards the prediction of compressive strength of high calcium fly ash-based GPC.

## 2. Literature Review

The ML learning algorithms are now widely used for the prediction of required outcomes. The mechanical properties of concrete are effectively forecasted via various ML techniques. The ANN, DT, SVM, GEP, and other ensemble ML approaches are most popular for the prediction of different properties of concrete. Iqbal et al. [46] used the GEP technique to predict the mechanical properties of green concrete containing waste foundry sand and represent the effective applications of the GEP model towards the prediction. Golafshani et al.’s [47] research was based on the ANN approach to forecasting the mechanical properties of sustainable concrete with waste foundry sand. It was reported that the ANN technique could be effectively used for the prediction of any type of concrete. Sun et al. [48] used the conventional neural network for the prediction of mechanical properties from microstructure images in the fiber-reinforced polymer. It was noted that the trained models could be applied to identify the position of potential damage site in the fiber-reinforced polymer. Akande et al.’s [49] study was based on the performance comparison of ANN and SVM algorithms towards the prediction of the compressive strength of concrete. The study represented that the SVM approach’s progress performance was slightly better compared to the ANN technique. The application of numerous types of ML approaches towards predicting many properties of concrete containing various types of industrial wastes is listed in Table 1.

## 3. Methodology and Data Description

**Figure 1:**
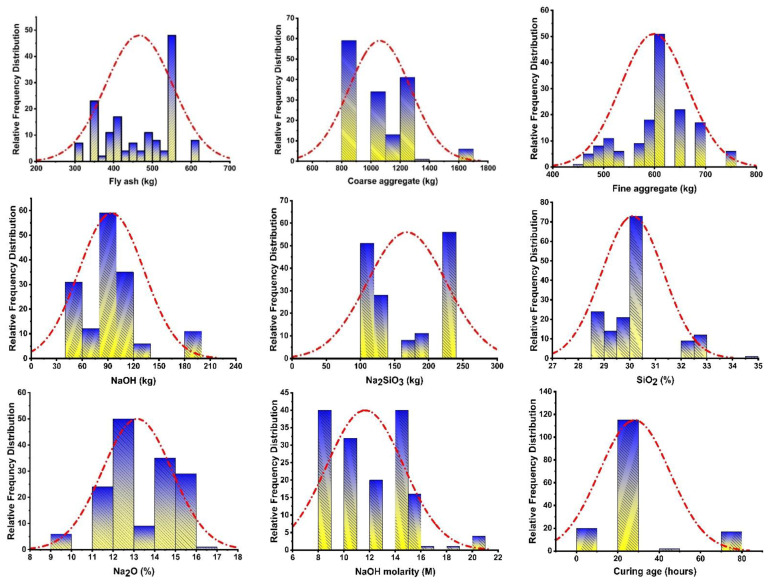
Relative frequency distribution of the input variables.

It is a fact that the supervised ML algorithms required a number of input parameters to run the model for the selected ML approach. The database is the key aspect for running the models to predict the required output. The data used in this research for running the models for both ensemble and individual ML techniques were obtained from the published literature [73,74,75,76,77,78,79,80,81]. The individual (ANN) and ensemble (AdaBoost and boosting) models were run based on the nine input parameters such as Na_2_SiO_3_, NaOH, SiO_2_, Na_2_O, the molarity of NaOH, and age of the curing to have the result in the form of output (CS). The performance of the models is also based on the number of input variables, which indicates that the input parameters have a significant effect on the accuracy of the models toward the prediction of their outcomes [82]. This research is also based on nine above-mentioned input variables with a total of 154 data points to forecast the outcome (CS) of high calcium fly ash GPC. The python coding was incorporated in the spyder (4.1.4) of the Anaconda software to run all the employed models. The coefficient correlation R^2^ value was the indication of an accuracy level between 0–1. The higher value of R^2^ and lower value of the errors (MAE, MSE, RMSE) indicates the high accuracy of the selected model towards the prediction of the required output. The descriptive statistics of the input variables used to run the model in the study can be seen in Table 2, while the relative frequency distribution of these variables is depicted in Figure 1.

### 3.1. Artificial Neural Network (ANN)

ANNs are also known as neural networks (NNs), and this is the computing system stimulate by the biological neural network, which accounts for animal brains. ANN is established on a group of nodes or units which are interconnected with one another, known as artificial neurons. The neurons’ structure and process are the reflections of the brain. These neurons receive a signal before processing and have the capability to signal the neuron which relates to it. The original number is a “signal” at a connection, and the outcome of every neuron is enumerated by other non-linear functions of the sum of its inputs. The connections are known as the edges. Edges, along with the neurons, normally have a weight that accommodates as learning proceeds. The increase and decrease of the weight are based on the strength of the signals at the connection. Neurons may have an entrance such as a signal processed only if the signal of the aggregate crosses that entrance. The neurons are typically arranged in the form of layers. Every layer has its own function on its outputs. These layers are the path for the signals which travel from the first layer (the input layer) to the last layer (the output layer), possibly after passing these layers the number of times. The same function and process are also adopted to predict the mechanical properties of concrete in civil engineering.

### 3.2. Boosting Algorithm

Boosting was initially invented by the theorists who were involved in computational learning, while machine learning researchers then generalized it. The boosting is also considered as one of the most used base learners. It is termed an ensemble algorithm which tends to a weighted average of predictions of individual classifiers. Boosting is also one of the powerful regression tools. Boosting can perform tasks when there are more variables than those of the given observation. This ensemble algorithm can split the given model into a number of sub-models to have a better value of coefficient correlation (R^2^). This ML approach is also used for the more accurate prediction of the required outcome. The mechanical properties of different types of concrete are being forecasted by employing the boosting technique at high accuracy. The other ensemble ML algorithms, along with the boosting, are also incorporated to predict the performance of concrete for comparison. Two parameters are required to explain the basic boosting machine learning approach. During the process, the number of splits (number of nodes) can be adjusted to fit each regression. The total number of nodes is equal to the sum of the splits multiplied by one. Identifying one split results in the creation of an additional model with only main effects. Identifying two splits corresponds to the model’s primary effects and two-way interconnections, respectively. Boosting operates in the same manner as other ensemble machine learning algorithms.

### 3.3. AdaBoost Algorithm

The ensemble technique is a machine learning concept that allows for the training of multiple models using a common learning algorithm. The ensemble consists of many algorithms collectively referred to as multi-classifiers. To resolve the issue, a group of hundreds or thousands of learners working toward a common goal comes together. AdaBoost is a supervised machine learning technique that makes use of ensemble learning. It is also known as adaptive boosting because the weights are re-assigned to each instance, with increased weights assigned to instances that were incorrectly classified. Boosting techniques are frequently used in supervised machine learning to reduce bias and variance. These ensemble techniques are used to strengthen the learner who is having difficulty. During the training phase for the input data, it employs an infinite number of DTs. While constructing the initial DT/model, high priority is placed on the recorded data that are incorrectly classified throughout the initial model. These data records are the only ones that are used as input for another model. The procedure outlined above will be repeated until the desired number of base learners is obtained. On binary classification problems, the AdaBoost regressor outperforms all other regressors in terms of improving the performance of DTs. Furthermore, it is used to improve the performance of other machine learning algorithms. It works especially well when used with a slow learner. Ensemble algorithms are most frequently used in the field of civil engineering, particularly for predicting the mechanical properties of concrete.

## 4. Results and Discussions

### 4.1. Statistical Results from Artificial Neural Network (ANN) Model

**Figure 2:**
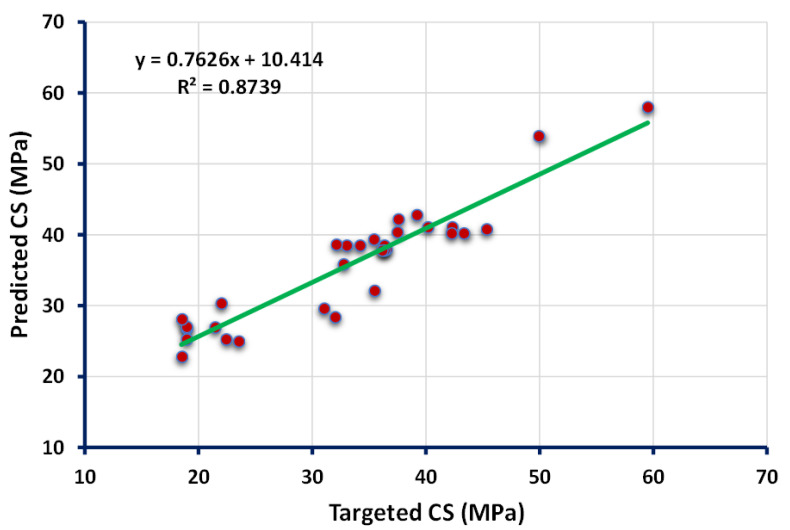
Relationship between the targeted and predicted results obtained from the ANN model.

**Figure 3:**
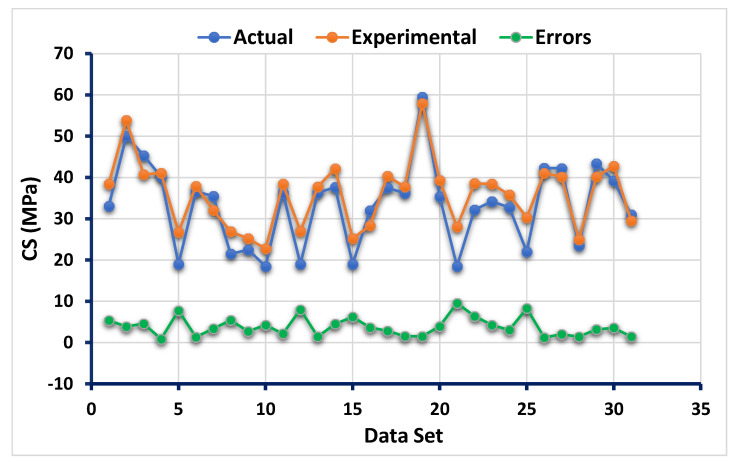
Error distribution of the targeted and predicted results from the ANN model.

The statistical findings from the ANN model between the targeted result obtained from the experimental work and forecasted outcome can be seen in Figure 2. The result of the ANN model reveals that the accuracy level was impressive towards the prediction of CS of flay ash-based GPC as indicated from the coefficient correlation (R^2^) value (0.87). However, the distribution of the errors from the actual and forecasted results is depicted in Figure 3. The errors’ maximum, minimum, and average values were 9.56 MPa, 0.85, and 3.86 MPa, respectively. Moreover, it was noted that 25.8% of the error data lie between 0 to 2 MPa, and 48.38% of this data was reported between 2 MPa to 5 MPa. However, only 19.35% of the errors data was observed to be above 5 MPa.

### 4.2. Statistical Results from Boosting Approach

**Figure 4:**
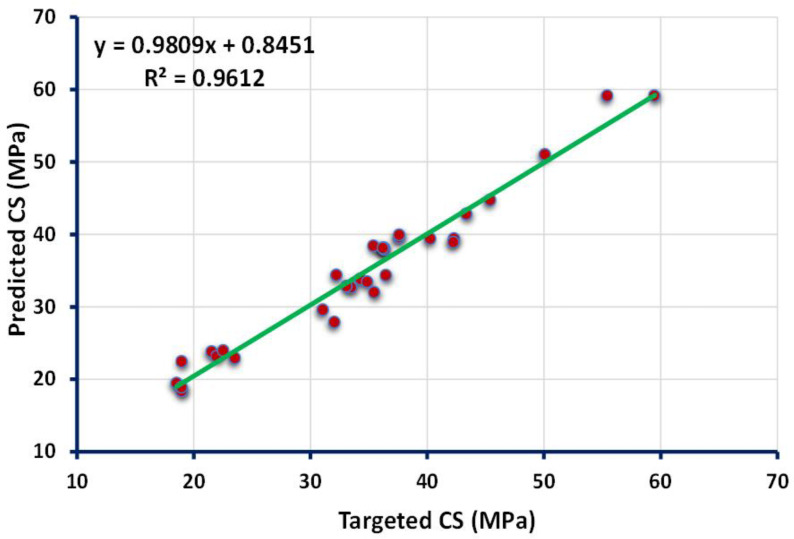
Relationship between the targeted and predicted results obtained from boosting approach.

**Figure 5:**
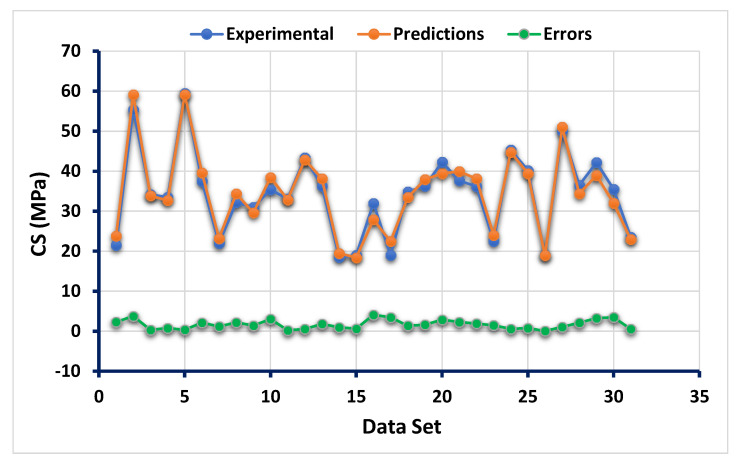
Error distribution of the targeted and predicted results from boosting approach.

The statistical analysis of the boosting technique indicates the strong, adamant relationship between the targeted output obtained from the experimental approach and the forecasted outcome given by the boosting regressor, as shown in Figure 4. The closer dot from the straight line is the indication of the high accuracy of the employed model. The reflection of the high value of the R^2^ value (0.96) confirms a more accurate model towards the prediction of CS of high calcium fly ash-based GPC. The indication of the distribution of errors from the predicted and actual data can be seen in Figure 5. The maximum, minimum, and average values of the error distribution for the model results obtained from the bagging technique were 4.08 MPa, 0.06 MPa, and 1.69 MPa, respectively. In addition, 35.48% of the total error data occurred between 0 to 1 MPa, while 45.16% of the data was reported between 1 MPa and 3 MPa. However, only 3.2% of the error data appeared above 4 MPa.

### 4.3. Statistical Result for Adaboost Approach

**Figure 6:**
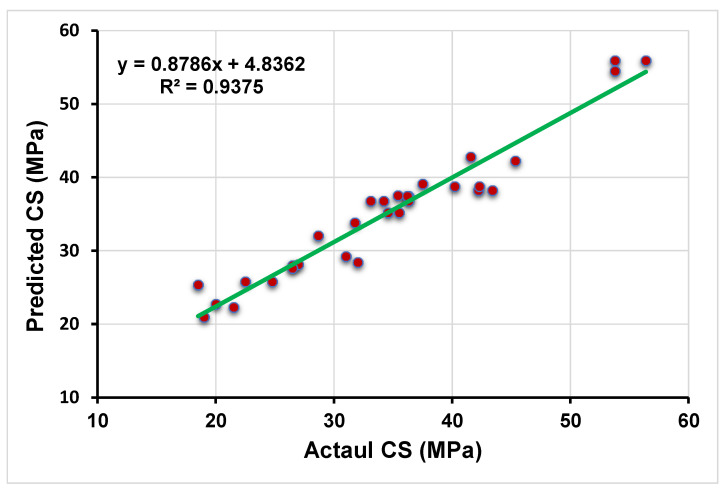
Relationship between the targeted and predicted results obtained from AdaBoost approach.

**Figure 7:**
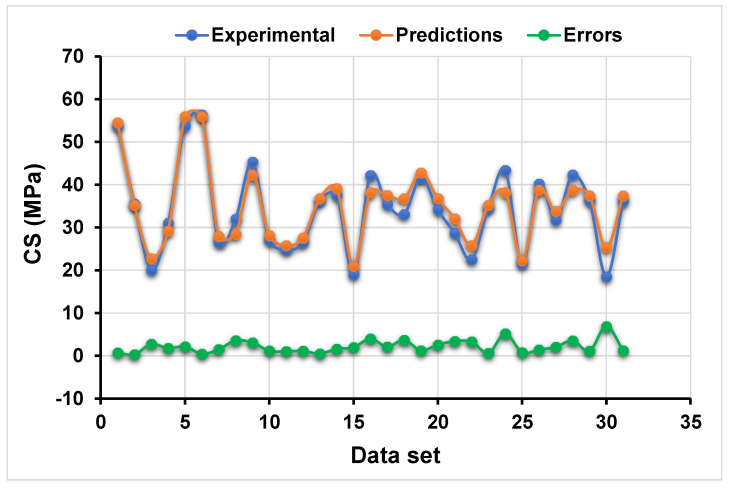
Error distribution of the targeted and predicted results from AdaBoost approach.

Figure 6 and Figure 7 compare the AdaBoost model’s actual and projected outputs. Figure 6 depicts the correlation between actual and projected results, which has an R^2^ value of 0.94, indicating that the R^2^ model is more precise than the ANN model and less accurate than the boosting model in terms of outcome precision. The distribution of actual values (targets), predicted values, and errors for the AdaBoost model is depicted in Figure 7. The error’s maximum, minimum, and average values were 6.87, 0.03, and 2.16 MPa, respectively. However, 19.35% of error values were less than 1 MPa, 51.61% were between 1 and 3 MPa, 22.58% were between 3 and 5 MPa, and only 6.45% were greater than 5 MPa. These lower error values also support the AdaBoost model’s greater accuracy when compared to the ANN model.

### 4.4. K-Fold Cross-Validation Process

The validity of the employed models was evaluated by incorporating the process of k-fold cross-validation. The approach of k-fold cross-validation is normally adopted for the evaluation of the model’s validity. This process gives the test and train data in the form of coefficient of determination (R^2^) and errors (MAE, MSE, RMSE). The higher value of R^2^ and lesser value of the errors indicate an accurate model towards the prediction. In this process, the database is randomly scattered and split into ten groups, from which the nine groups were used for training while one group was allocated for validation purposes. The repetition of the said process took ten steps to have an appreciable result. The execution of this process for the model lead towards high accuracy. 80% of the database is assigned to train the employed models, while the remaining 20% of the data set is allocated for testing the models. However, the statistical evaluation of the errors (MAE, MSE, RMSE) for both the models is listed in Table 3. The statistical results from both models’ data clearly show that the boosting approach had fewer error values than the ANN model. The following Equations (1)–(3) in accordance with the literature [46,57] were used to evaluate the response of each parameter.

(1) $$\mathrm{MAE}=\frac{1}{n}\overset{n}{\underset{i=1}{\sum }}\left|x_{i}-x\right|$$

(2) $$\mathrm{MSE}=\frac{1}{n}\overset{n}{\underset{i=1}{\sum }}{\left(y_{pred}-y_{ref}\right)}^{2}$$

(3) $$\mathrm{RMSE}=\sqrt{\sum \frac{{\left(y_{pred}-y_{ref}\right)}^{2}}{n}}$$

where: *n* = total number of data samples, x, $y_{ref}$ = reference values in the data sample, $x_{i}$, $y_{pred}$ = predicted values from models.

**Figure 8:**
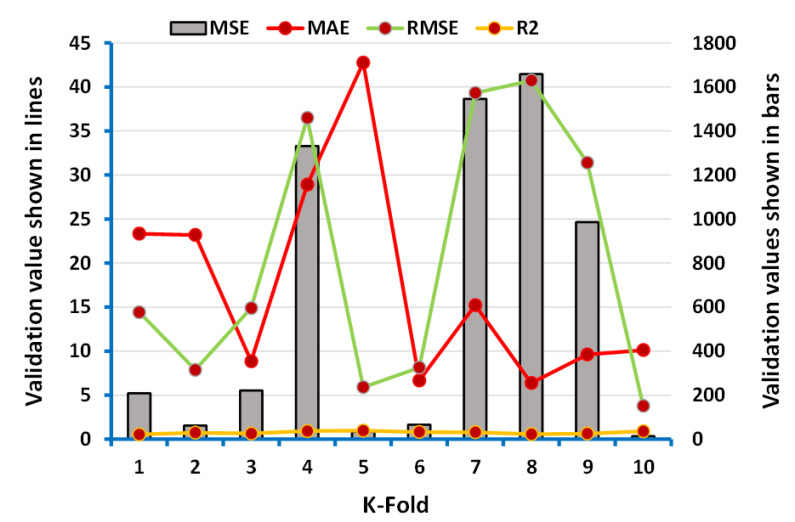
Representation of the statistics for k-fold cross-validation of the ANN model.

**Figure 9:**
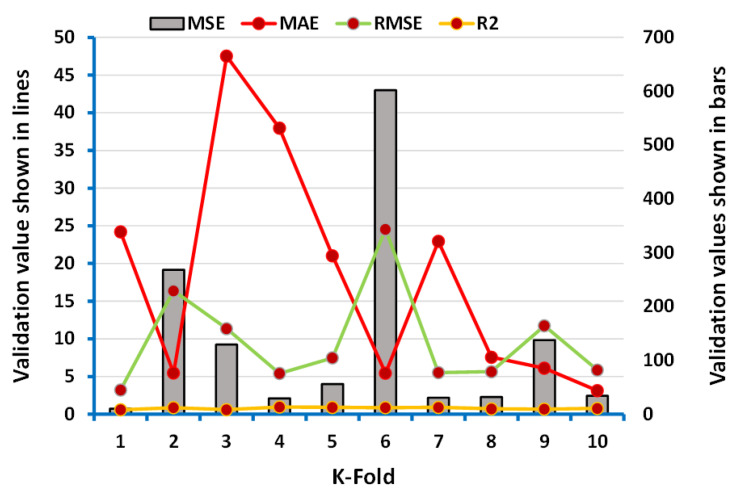
Representation of the statistics for k-fold cross-validation of the boosting model.

The data of the parameters (R^2^, MAE, MSE, and RMSE) obtained from the data of employed algorithms (ANN, boosting) were taken for the k-fold cross-validation process as depicted in Figure 8 and Figure 9. However, the maximum, minimum, and average values of MSE during the process of k-fold cross-validation for ANN’s model were 1658.28 MPa, 14.33 MPa, and 612.97 MPa, respectively, as shown in Figure 8. In addition, the maximum values of MAE, RMSE, and R^2^ were noted as 42.76 MPa, 40.72 MPa, and 0.98, respectively. In comparison, the maximum, minimum, and average results of MSE for boosting approach were 601.90 MPa, 10.41 MPa, and 132.96 MPa, respectively, and can be seen in Figure 9. However, the maximum values reported for MAE, RMSE, and R^2^ were 47.51 MPa, 24.53 MPa, and 0.95, respectively.

## 5. Sensitivity Analyses

**Figure 10:**
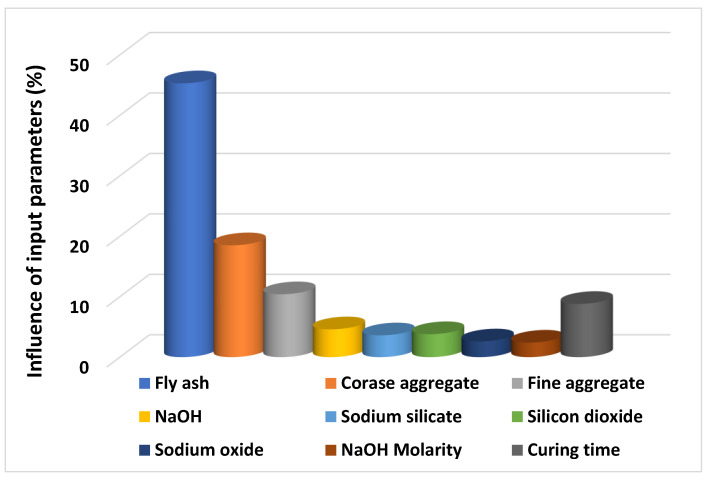
Influence of input parameters towards the prediction of outcome (CS) of GPC.

To check the influence of each variable towards the prediction of CS of high calcium fly-ash-based GPC, the analysis was carried out known as the sensitivity analysis. Since the input parameters play a key role in the accuracy of employed models for the prediction aspect, it is also necessary to know the effect of input parameters individually on the predicted outcome. The contribution of each input parameter towards the forecasted output of the CS of GPC can be seen in the Figure 10. It was noted that the important parameters fly ash (45.3%), coarse aggregate (18.5%), and fine aggregate (10.4%) have contributed significantly towards the prediction of the strength property of GPC. In addition, the other input variables NaOH, Na_2_SiO_3_, SiO_2_, Na_2_O, NaOH molarity, and curing time, contributed the least for the forecasting of CS of GPC. The following Equations (4) and (5) retrieved from the literature [41] were used to calculate the contribution of each variable towards the predicted outcome.

(4) $$N_{i}={\;f}_{\max}\left(x_{i}\right)-{\;f}_{\min}\left(x_{i}\right)$$

(5) $$S_{i}=\frac{N_{i}}{\sum_{j-i}^{n}N_{j}}$$

where f_max_ (x_i_) and f_min_ (x_i_) are the maximum and minimum of the estimated output over the ith output.

## 6. Discussion

**Figure 11:**
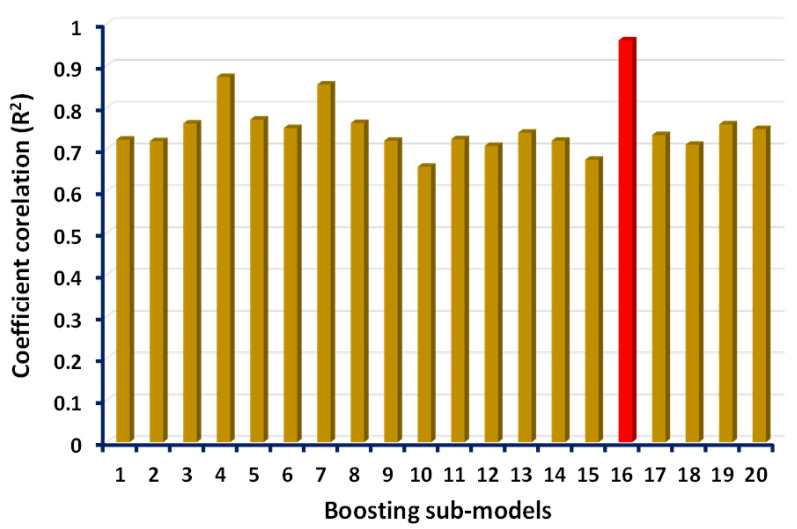
Sub-models of the boosting approach indicating the coefficient correlation (R^2^).

This research describes the effect of supervised ML algorithms in terms of prediction for the mechanical property (CS) of GPC containing high calcium fly ash. Three supervised ML algorithms—ANN, AdaBoost, and boosting approaches—were introduced to forecast the required outcome. To obtain the single output of compressive strength total of nine input variables were incorporated. The python coding was the key aspect for running the employed models in the anaconda software. The descriptive statistical analysis was also carried out for the input parameters to check the ranges, mode, median, standard deviation, and other relevant information. The results indicate that the ensemble ML algorithms (boosting and AdaBoost) were more effective when compared with the result of the individual (ANN) model’s outcome. In comparison, the highest value of the coefficient of determination (R^2^) of boosting technique (0.96) is the indication of its better performance towards the prediction of the outcome as opposed to the R^2^ value of ANN and AdaBoost model, which were reported as 0.87 and 0.93, respectively. The accuracy of the ensemble ML technique is because of its adopted procedure of splitting the model into sub-models, and the result of the sub-models of boosting technique can be seen in the Figure 11. The 16th sub-model shows the highest R^2^ value, the data of which was then incorporated for further evaluation. Based on the obtained data, the statistical evaluation of the errors (MAE, MSE, RMSE) was performed for the confirmation of the model’s accuracy. Moreover, the k-fold cross-validation process was also included in the research to confirms the precision of the employed models. In addition, the sensitivity analysis reveals that the fly ash (45.3%) and sodium hydro oxide (18.5%) contributed the most towards the prediction of compressive strength of GPC, while another contribution was reported least for the prediction of output.

## 7. Conclusions

This study explains the performance comparison of the ensemble ML approaches (boosting and AdaBoost) and individual ML techniques (ANN) to forecast the CS of geopolymer concrete containing high calcium fly ash. The mechanical property (CS) of GPC was successfully predicted at a relatively high accuracy when compared to the actual result. The coefficient of determination and the result of various errors along with the statistical checks were incorporated to evaluate the performance comparison of the employed ML approaches. The following conclusions from the study can be drawn:

- The ML algorithms (both ensemble and individual) can be successfully utilized to predict the mechanical properties of any type of geopolymer concrete.
- The ensemble ML techniques boosting and AdaBoost were very effective when treated for forecasting the CS of GPC by indicating the high value of R^2^ equals 0.96 and 0.93, respectively. However, the individual ML approach (ANN) gives the R^2^ value equal to 0.87, indicating the poor accuracy level towards the prediction of CS as opposed to boosting algorithm.
- The high precision level of the boosting technique also confirms the lesser values of the errors from the ANN approach. The MAE, MSE, and RMSE values for boosting were 1.69 MPa, 4.16 MPa, and 2.04 MPa, respectively, while these values for the ANN model were 3.86 MPa, 20.16 MPa, and 4.49 MPa, respectively, and similar values were reported for AdaBoost model as 2.16 MPa, 6.84 MPa, and 2.62 MPa, respectively.
- Statistical analyses and method of k-fold cross validation also confirm that the performance of boosting ML technique was effective to forecast the CS as compared to the ANN model.
- Sensitivity analysis reveals that the fly ash was the superior parameter which contributed magnificently at 45.3% towards the prediction of CS for GPC.
- Overall, the combined effect of the obtained result from the coefficient of determination (R^2^) and result from various errors makes an indication for boosting technique as the best performer when compared to AdaBoost and ANN model.

It has been reported and recommended that ensemble ML techniques can be successfully employed to forecast the mechanical properties of any type of concrete with accurate results. To obtain more accurate results for the selected ML algorithms, the number of databases can be increased via an experimental approach in the laboratory. Another aspect of enhancing the accuracy level of the ML approach is to increase the number of input parameters such as the temperature effect, additional ages of the sample’s strength, etc. The application of ML techniques to predict the required outcomes in the field of civil engineering not only reduces the cost of the projects and minimizes the time required to complete the tasks.

## Figures and Tables

**Table 1:** Prediction properties details via supervised machine learning techniques.

Sr. No	Type of ML	Notation	Data Points	Forecasted Properties	Year	Material Used	References
1.	Support vector machine	SVM	144	CS	2021	FA	[50]
2.	Gene expression programming	GEP	303	Bearing capacity of concrete-filled steel tube column	2019	_	[51]
3.	Data Envelopment Analysis	DEA	114	CS Slump test L-box test V-funnel test	2021	FA	[52]
4.	Gene expression programming, Artificial neural network, Decision tree	GEP, ANN, DT	642	Surface Chloride Concentration	2021	FA	[53]
5.	Support vector machine	SVM	-	CS	2020	FA	[54]
6.	Support vector machine	SVM	115	Slump test L-box test V-funnel test CS	2020	FA	[55]
7.	Gene Expression Programming	GEP	351	CS	2020	GGBS	[56]
8.	Gene Expression Programming	GEP	54	CS	2019	NZ (Natural Zeolite)	[57]
9.	Gene Expression programming	GEP	357	CS	2020	-	[44]
10.	Random forest and Gene Expression programming	RF and GEP	357	CS	2020	-	[57]
11.	Artificial neuron network	ANN	205	CS	2019	FA GGBFS SF RHA	[58]
12.	Intelligent rule-based enhanced multiclass support vector machine and fuzzy rules	IREMSVM-FR with RSM	114	CS	2019	FA	[59]
13.	Random forest	RF	131	CS	2019	FA GGBFS FA	[60]
14.	Multivariate Adaptive regression spline	M5 MARS	114	CS Slump test L-box test V-funnel test	2018	FA	[61]
15.	Random Kitchen Sink Algorithm	RKSA	40	V-funnel test J-ring test Slump test CS	2018	FA	[62]
16.	Adaptive neuro-fuzzy inference system	ANFIS	55	CS	2018	-	[63]
17.	Artificial neuron network	ANN	114	CS	2017	FA	[64]
18.	Artificial neuron network	ANN	69	CS	2017	FA	[65]
19.	Individual and ensemble algorithm	GEP, DT and Bagging	270	CS	2021	FA	[66]
20.	Individual with ensemble modeling	ANN, bagging and boosting	1030	CS	2021	FA	[67]
21.	Multivariate	MV	21	CS	2020	Crumb rubber with SF	[68]
22.	Gene Expression programming	GEP	277	Axial capacity	2020	-	[69]
23.	Adaptive neuro-fuzzy inference system	ANFIS with ANN	7	CS	2020	POFA	[70]
24.	Response Surface Method, Gene expression programming	RSM, GEP	108	CS	2020	Steel Fibers	[71]
25.	Decision tree, artificial neural network, bagging, and gradient boosting	DT, ANN, BR, GB	207	CS	2021	FA	[72]

**Table 2:** Descriptive analysis of the input variables.

Parameters	Fly Ash	Coarse Aggregate	Fine Aggregate	NaOH	Na_2_SiO_3_	SiO_2_	Na_2_O	Molarity of NaOH	Curing Age
Mean	465.79	1060.99	598.93	94.26	167.87	30.12	13.16	11.65	28.13
Standard Error	6.97	16.93	5.29	3.07	4.64	0.10	0.13	0.24	1.37
Median	494.00	1091.00	600.00	95.00	138.00	30.00	12.00	12.00	24.00
Mode	550.00	838.00	600.00	95.00	239.00	30.00	12.00	8.00	24.00
Standard Deviation	86.54	210.13	65.61	38.05	57.61	1.20	1.67	2.98	17.01
Sample Variance	7489.66	44,152.69	4305.08	1447.46	3318.74	1.43	2.79	8.90	289.26
Kurtosis	−1.26	0.77	−0.07	1.78	−1.77	1.92	−0.68	−0.31	2.44
Skewness	−0.26	0.80	0.01	1.02	0.20	1.50	−0.28	0.42	1.67
Range	300.00	846.00	291.00	157.00	136.00	6.00	7.20	12.00	69.00
Minimum	300.00	838.00	459.00	41.00	103.00	28.70	9.00	8.00	3.00
Maximum	600.00	1684.00	750.00	198.00	239.00	34.70	16.20	20.00	72.00
Sum	71,732.00	163,393.00	92,235.40	14,516.20	25,851.42	4637.90	2026.20	1794.00	4332.00
Count	154.00	154.00	154.00	154.00	154.00	154.00	154.00	154.00	154.00

**Table 3:** Statistical checks for employed algorithms.

ML Algorithms	MAE (MPa)	MSE (MPa)	RMSE (MPa)
Artificial neural network (ANN) model	3.86	20.16	4.49
Boosting model	1.69	4.16	2.04
AdaBoost model	2.16	6.84	2.62

## Data Availability

The data presented in this article are available within the article.
